# Tungiasis-associated morbidity in pigs and dogs in endemic villages of Uganda

**DOI:** 10.1186/s13071-016-1320-0

**Published:** 2016-01-27

**Authors:** Francis Mutebi, Jürgen Krücken, Hermann Feldmeier, Charles Waiswa, Norbert Mencke, Georg von Samson-Himmelstjerna

**Affiliations:** School of Veterinary Medicine and Animal Resources, College of Veterinary Medicine, Animal Resources and Biosecurity, Makerere University, P.O. box 7062, Kampala, Uganda; Institute for Parasitology and Tropical Veterinary Medicine, Freie Universität Berlin, 14163 Berlin, Germany; Institute of Microbiology and Hygiene, Charité University Medicine, Berlin Campus Benjamin Franklin, Hindenburgdamm 30, 12203 Berlin, Germany; Bayer Animal Health, 51368 Leverkusen, Germany

**Keywords:** *Tunga penetrans*, Tungiasis, Clinical pathology, Dogs, Pigs, Uganda

## Abstract

**Background:**

*Tunga penetrans* (Insecta, Siphonaptera, Tungidae) causes severe morbidity among heavily infected humans and animals in Latin America and sub-Saharan Africa. The clinical pathology of tungiasis in animals has never been studied systematically.

**Methods:**

This was a cross-sectional study conducted between January to March 2015, aimed at describing tungiasis-associated clinical pathology in 121 and 20 *T. penetrans*-infected pigs and dogs, living in nine and five endemic rural villages respectively located in Bugiri District, Busoga, Uganda.

**Results:**

The parasite load of infected animals ranged from one to 246 (median 8) and one to eight (median 2) in pigs and dogs, respectively. In pigs 99.3 % and in dogs 100 % of the lesions were located on feet. In pigs, hind legs were significantly more affected than front legs (90.9 % vs. 57.9 %; *p* = 0.002) and also had more lesions than the front legs (median 5 vs. 1; *p* = 0.0001). However, in dogs localization of lesions between front and hind legs never differed significantly (front, 50 % vs. hind, 65 %; *p* = 0.51) and so were the number of lesions (median front = 0.5 vs. median hind = 2; *p* = 0.7). Acute and chronic clinical pathology coexisted. The most common disease manifestations in pigs were hoof wall erosions (68.6 %), tissue necrosis of hoof wall and skin (66.1), pain at infection sites (47.9 %), hoof deformity (45.5 %), fissures (44.6 %) and edema (44.6 %). In dogs, tungiasis mainly presented with pain at attachment site (80 %), ulcers (55 %), necrosis (30 %) as well as hyperemia and edema (both 15 %). One pig had lost dew claws while two had loose detaching claws. Despite a lower number of sand fleas, a higher proportion of infected dogs (20 %) than pigs (5.8 %) exhibited functional limb use difficulties (*p* = 0.05).

**Conclusions:**

The pattern of clinical manifestations in pigs and dogs were very similar to those reported from affected humans and rats. The important morbidity associated with animal tungiasis makes the disease a serious veterinary health problem in sub-Saharan Africa warranting treatment and control for optimal animal production.

## Background

Tungiasis is a zoonosis caused by female sand fleas of the species *Tunga penetrans* and *Tunga trimamillata* embedded in the skin. It is a Neglected Tropical Disease which has re-emerged in epidemic dimensions in East Africa in the last decade [1–4]. While *T. penetrans* is endemic in many countries of South America, the Caribbean Islands and sub-Saharan Africa, *T. trimamillata* has been reported in Peru, Ecuador and Brazil [5]. Tungiasis is often reported in non-endemic regions among tourists and immigrants from endemic areas [6–8]. The disease in humans has been described in detail and is associated with important morbidity [2, 4, 9, 10] whose severity increases with intensity of infection [11].

Tungiasis also occurs in a wide range of domestic, peri-domestic and wild animals [12–14] usually alongside human tungiasis in endemic communities of South America and sub-Saharan Africa [12–15]. With the increasing number of international journeys with pets, animal tungiasis is also being detected in non-endemic countries [16]. In animals, sand fleas mainly localize on legs but also infect other organs which often come into contact with the ground, for example; mammary glands, gonads, muzzle and the tail [12, 17–20]. Limb infections cause variable degrees of lameness [13] and mammary gland infections have been reported to cause agalactia among lactating sows [17, 18].

Systemic studies on morbidity in animals do not exist and, hence, clinical pathology and economic significance of animal tungiasis are largely unknown among veterinary practitioners. Consequently diagnosis and management of animal tungiasis remain a challenge. The situation is worsened by the fact that tungiasis predominantly occurs in marginalized populations [21] and is associated with poverty; hence, it exists in situations where veterinary services are limited.

Pigs and dogs have been reported as the major domestic animal hosts of zoonotic sand fleas in endemic countries [13–15]. In the study area, the odds of occurrence of animal tungiasis in a household increased with presence of human tungiasis, presence of pigs, with the number of animal species kept and with the size of the homestead [14]. This study aimed to systematically document the clinical pathology associated with sand flea infections in pigs and dogs in endemic villages in Uganda.

## Methods

### Study area

The study area has been described before in detail [14]. Briefly the study was carried out in 10 neighboring rural tungiasis-endemic villages from three parishes of Bulidha sub county, Bugiri District, South Eastern Uganda which were purposively selected because they were known to have a high prevalence of human tungiasis. The villages sampled were Makoma 1, Masolya, Busindha, Isakabisolo, Matyama, Busakira, Namungodi, Busanoin Makoma Parish; Nagongera in Bulidha Parish and Kibuye in Wakawaka Parish. The area has two rainy seasons which support subsistence arable agriculture among the communities. All the roads and paths in the villages are made of murram and over 95 % of the households have non-concrete floors. Animal rearing is also on subsistence scale, farmer education services are very limited and parasite control among animals is practiced only occasionally by a few farmers. Livestock stay on earthen grounds with no formal housing infrastructures. While domestic ruminants such as goats, sheep and cattle are tethered most of the time, pigs are only confined intermittently during the crop growing season and released most of the time during the dry season. Dogs, cats and all poultry are left to roam freely throughout the year.

### Study design and study population

The principal study design has been described previously [14]. In brief, this was a cross sectional study performed from January 23 to March 25, 2014 which coincided with the middle to the end of the dry season when tungiasis is particularly abundant. All pigs and dogs in the 10 villages which had stayed in the area for at least four weeks and those born in the area (but below four weeks of age) were included in the study if their owners consented. Tungiasis-associated morbidity was described for all pigs and dogs with at least one lesion.

### Clinical examination of pigs and dogs

All pigs were restrained physically but after temporal physical handling by the owner, some dogs were sedated with xylazine (0.5 mg/kg body weight intramuscularly) and ketamine (7 mg/kg body weight intramuscularly) used with atropine (0.02 mg/kg body weight intramuscularly) to control vomiting. The whole body was examined systematically from the head, trunk, tail, ventral abdomen and limbs by parting the hairs, palpation and observation for tungiasis-associated lesions. Emphasis was put on the predilection sites of sand fleas, i.e. legs, tail, mammary glands and the testes [5, 17–20]. The hooves of the pigs and paws of dogs were washed using a brush and water to permit efficient detection of the embedded sand fleas. Lesions were staged according to the Fortaleza classification [9], counted and documented by description and photography.

All parasitological and biographical information of infected animals obtained by animal examinations, observations and information from animal owners were recorded on standardized forms. Since there were no animal records, age and management related information was obtained by interviewing animal owners. Furthermore, body condition score, breed and weight among other related information were obtained by observation and physical examination. The body condition scores were determined using the systems described by Kimberly and associates [22] and Defra [23] for dogs and pigs, respectively. Weight of pigs was estimated using a girth tape.

Staging of *T. penetrans* lesions was based on the dynamic features of tungiasis as described before [9]. Viable lesions of *T.penetrans* presented as a dark spot with or without pain surrounded by a hyperemic zone (stage 2) or as glassy nodular patches of 3–12 mm in diameter, with a central dark spot which exuded eggs or watery fluid on a gentle touch (stage 3). Non-vital lesions were identified as either brown to black slightly raised patches surrounded by a necrotic area (stage 4) or as epidermal circular craters with or without necrotic tissue edges (stage 5).

All photographs of the lesions from all *T. penetrans*-infected pigs and dogs were reviewed daily to understand the morphological alterations at different topographic sites. In any animal with at least one lesion, irrespective of size and severity, the clinical pathology was documented. Only morphological changes in close proximity (≤5 mm) of the embedded sand fleas were attributed to tungiasis. A cluster of lesions was defined as ≥3 lesions which were not more than 5 mm apart from each other.

For morphological identification of sand flea species at the College of Veterinary Medicine, Animal Resources and Bio-security (CoVAB), ten and five sand fleas were extracted from 10 pigs and three dogs, respectively. Fleas were preserved in 10 % ethanol in the field before morphological analysis in the laboratory. Identification of the embedded females was based on features as described before [5, 24]. All extracted sand fleas exhibited a clover leaf-like appearance of the chitin of the anterior neosomic abdomen when viewed cranially and hence were identified as *T.penetrans* [14].

### Statistical analysis

Data was entered into excel sheets (Microsoft Office, 2007) and validated by checking all entries against all of the original data collection forms. Then data was exported to Stata^®^ Software package, Version 13 (Stata Corporation, College Station, Texas 77845 USA, stata@stata.com) for analysis. Spearman’s rank correlation coefficient was calculated to assess the relationship between pairs of continuous groups of variables which were not normally distributed. The Wilcoxon rank sum test was used to establish if there were differences in the number of lesions in animals between two groups or predilection sites. Mid p-exact tests were conducted using the rate2by2.test function as implemented in the epitools package in R-software to establish the significance of differences between proportions. Only *p*-values of less than 0.05 were considered statistically significant.

### Ethical considerations

Study protocols were considered and ethical clearance was granted by the College of Veterinary Medicine, Animal Resources and Bio-security (COVAB) (Ref. VAB/REC/14/101) and the National Council of Science and Technology (Ref.1621). The study was conducted in collaboration with the responsible local Veterinary Extension Officers. Participation was optional and animal owners provided informed written consent. In addition, affected households were briefed on the biology of *T. penetrans* and an insecticidal wound spray (Supona^®^ aerosol, Pfizer Laboratories (Pty) Ltd, South Africa) was applied on affected sites and associated wounds of animals.

## Results

### Animal information and management practices

A total of 514 out of 524 (98.1 %) pigs from 155 of the 158 (98.1 %) households with pigs were examined in the 10 villages [14] while 282 out of 299 (94.3 %) dogs from 120 out of 121 (99.2 %) households with dogs were examined [14]. Of the 514 pigs, 121 (23.5 %, 95 % CI = 20.0 %–27.5 %) from 54 households (34.8 %, 95 % CI = 27.5 %–43 %) and nine villages had tungiasis. On the other hand, 20 dogs (7.1 %, 95 % CI = 4.5 %–10.9 %) out of 282 from 15 (12.5 %, 95 % CI = 7.4 %–20.1 %) households and five villages had at least one lesion of tungiasis [14]. The findings presented are based on all the 121 pigs and 20 dogs with tungiasis from nine and five study villages, respectively. The prevalence of tungiasis was significantly higher in pigs than dogs (*p* <0.0001) [14].

All affected pigs were kept on non concrete floors either in an open area under trees/shrubs (113; 93.4 %) or non-roofed enclosures made of sticks or mud block walls erected under trees (8; 6.6 %) located at a distance of 0–50 m away from human compounds. With the exception of only three pigs, even other non-infected pigs which were examined were kept in a similar manner [14]. Other biographic information of infected pigs is presented in Table 1. All affected dogs were of the local breed (Basenji) with a median age of six months (range one week-48 months). Of the 20 infected dogs, 10 (50 %) were male and the others were female. The body condition scores of dogs varied from 2.5-4 (median = 3).

**Table 1:** Demographic information of pigs with tungiasis (*n* = 121)

Parameter	Results
Breed: Number (%)	
Mixed breeds	118 (97.5)
Exotic (Large white)	03 (2.5)
Age in months: median (range)	5 (0.5–30)
Sex: Number (%)	
Female	83 (68.6)
Male	38 (31.4)
Body weight in kg: median (Range)	123 (3–222)
Body condition score using a scale of 1–5: median (range)	3.4 (2–5)

### Topographic localization of sand fleas and parasite load

The 121 pigs had a total of 3357 lesions. However, the number of lesions per pig was highly variable: 47 (38.8 %) had light infections (1–4 lesions), 44 (36.4 %) had a moderate intensity (5–30 lesions) while 30 (24.8 %) had a heavy parasite load (>30 lesions). The lesions in the later group constituted 79 % of the total number of lesions detected. In contrast, dogs had few sand flea lesions and their number did not vary greatly. The 20 dogs had a total of 53 lesions. Of these, only two had 5–8 lesions while the other 18 had light infections (1–4 lesions).

Most of the sand flea lesions in pigs were localised on the legs (3333, 99.3 %), predominantly on the coronary band. Only 24 lesions (0.7 %) were located on the testes and the perineum (Table 2). Out of the 3333 lesions on the legs of pigs, 3318 (99.6 %) were localised on the coronary band and bulb of the claws and only 15 (0.4 %) were on the skin along metatarsal shafts. In two pigs, a single sand flea burrowed under the horny hoof wall at the coronary band of each of the animals.

**Table 2:** Topographic localization of sand flea lesions in pigs and dogs

Localization	Number of animals (%)	Number of lesions		
		Total	%	Median (range)
Pigs (*n* = 121)				
Legs	121 (100)	3333	99.3	8 (1–246)
Ectopic sites	2 (1.7)	24	0.7	0
Scrotum	1 (0.83)	14	0.42	0
Perineum	1 (0.83)	10	0.3	0
Front legs	70 (57.9)	756	22.5	1 (0–93)
Hind legs	110 (90.9)	2577	76.8	5 (0–154)
Accessory digits	73 (60.3)	1059	31.6	2 (0–117)
Principal digits	115 (95.0)	2259	67.3	5 (0–135)
Metatarsal shaft skin	1 (0.83)	15	0.45	0
Dogs (*n* = 20)				
Legs	20 (100)	53	100	2 (1–8)
Front legs	10 (50)	26	49.1	0.5 (0–5)
Hind legs	13 (65)	27	50.9	2 (0–5)
Digital foot pads	16 (80)	39	73.6	2 (0–5)
Metacarpal foot pads	2 (10)	2	3.8	0 (0–1)
Metatarsal foot pads	4 (20)	10	18.9	0 (0–4)
Carpal foot pads	1 (5)	2	3.8	0 (0–2)
Periungual area of toes	12 (60)	32	60.4	2 (0–5)
Sides of foot pad	9 (45)	16	30.2	0 (0–4)
Sole of foot pads	4 (20)	4	7.6	0 (0–1)
Interdigital space	1 (5)	1	1.9	0 (0–1)

Clustering of lesions (three or more lesions ≤ 5 mm apart) occurred in 57 (47.1 % of the affected pigs. Up to eight infection sites were found per pig with a very strong positive correlation between number of affected topographic sites and the total number of sand fleas (Spearman’s rho = 0.92, *p* = 0.0001). Lesion clusters were detected in six (30 %) dogs. Embedded sand fleas were observed at up to four different sites and the number of attachment sites also increased with the number of embedded sand fleas (Spearman’s rho = 0.71, *p* = 0.0005).

The hind legs in pigs were more frequently affected than front legs (*n* = 110, 90.9 % vs. *n* = 70, 57.9 % respectively, *p* = 0.002). Also, significantly more pigs had lesions on principal digits than accessory digits (*n* = 115, 95 % vs. *n* = 73, 60.3 % respectively, *p* = 0.0005). In contrast, in dogs the localization of lesions did not differ significantly between the front and hind legs (*n* = 10, 50 % vs. *n* = 13, 65 % respectively, *p* = 0.51). The number of dogs with lesions on the digital foot pads was also significantly higher compared to those with lesions on the metacarpal (*p* < 0.0003), carpal (*p* < 0.0002) and metatarsal foot pads (*p* = 0.0001). As regards actual localization site, most dogs had lesions on the periungual areas of the toes and the sides of the pads compared to the sole and inter digital spaces as shown in Table 3. The number of dogs never differed significantly between those with embedded sand fleas at periungual sites and the sides of the soles (*p* = 0.4) but fewer dogs had lesions on soles of foot pads (*p* = 0.005) and inter digital spaces (*p* <0.0001) than the periungual areas. Those with lesions in the footpad soles were only puppies. Although, the soles directly touch the ground, they had significantly smaller number of lesions than other sites (*p* < 0.0001).

**Table 3:** Clinical pathology of tungiasis in pigs and dogs

Clinical pathology	Number affected	Percentage (%)
Pigs (*n* = 121)		
Pain on digital pressure at affected site	58	47.9
Hyperemia	52	43.0
Edema (around lesions or entire digit)	54	44.6
Skin ulcers	38	31.4
Fissures	54	44.6
Necrosis and sloughing of skin or hoof wall	80	66.1
Hyperkeratosis	28	23.1
Loosening/detachment of digital and/or accessory claws	2	1.7
Loss of dew claws	1	0.8
Hoof wall erosion	83	68.6
Deformity	55	45.5
Overgrowth and lateral deviation of dew claws	21	17.4
Principal digital hoof overgrowth and lateral deviation	2	1.7
Hoof wall corrugations and roughening	48	39.7
Demelanisation	1	0.8
Anaemia	5	4.1
Dogs (*n* = 20)		
Pain on digital pressure at affected site	16	80
Hyperemia	3	15
Edema	3	15
Ulcers	11	55
Necrosis and sloughing of skin	6	30
Hyperkeratosis	1	5

For pigs, the number of embedded sand fleas on the hind legs was significantly higher than that on the front legs (median 5 and 1 respectively, *p* = 0.0001) but there was no such difference among infected dogs (median 2 vs. 0.5 respectively, *p* = 0.70). The difference in the number of embedded sand fleas between the principal and accessory digits of pigs was statistically significant (median 5 and 2, respectively; *p* = 0.0001). For dogs, digital foot pads had significantly more lesions than other foot pads combined (median 2 and 0 respectively, *p* < 0.0001). Also, the periungual sites (area around the base of claws) had more lesions compared to foot pad sides, soles and the inter-digital space combined (median 2 and 0 respectively, *p* = 0.0003).

### Clinical pathology

The main clinical findings detected in *T. penetrans*-infected pigs and dogs are shown in Table 3. Five pigs (4.1 %) but no dog had gross features of anaemia (pale mucous membranes). Seven pigs (5.8 %) of variable ages and four one week old puppies (20 %) had limb use difficulties characterised by frequent intermittent recumbence and limping while moving.

**Fig. 1:**
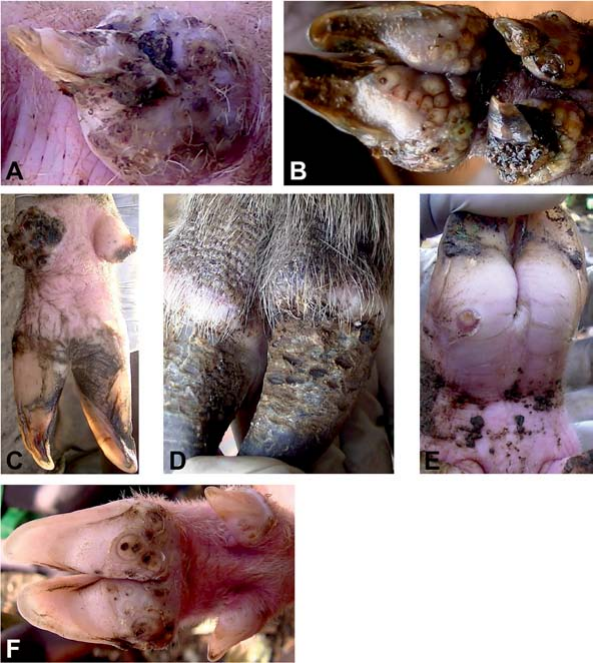
Clinical pathology of tungiasis in pigs. **a** Dew claw edema and horny wall surface erosions. **b** Clustering of sand fleas at the coronary band and extensive hoof wall necrosis. **c** Bilateral loss of dew claws in a case of heavy infection and overgrowth of digital claws. **d** Hyperkeratosis of the skin and fissures at the coronary band coupled with hoof wall erosions. **e** Intense rim of hyperemia around an embedded lesion at the coronary band. **f** Sand flea lesions on principal and accessory digits of a pig at various stages of development. Note the hyperemia around the lesions and the relatively large size of the lesions

**Fig. 2:**
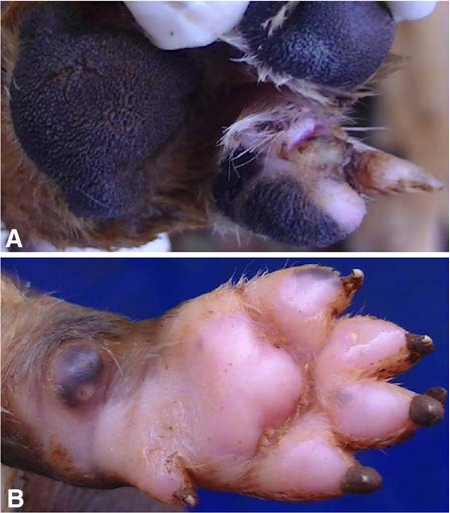
**a** Exteriorised sand flea lesions at the base of claw with a rim of hyperemia in an 18 month old dog. **b** Two sand fleas at the carpal foot pad of a one week old puppy surrounded by a zone of hyperemia

Representative clinical presentations are shown in Fig. 1 for pigs and Fig. 2 for dogs respectively. Edema due to tungiasis was entirely localised at the affected digit either as focal edema around individual lesions or few lesions in close proximity and at times involving the entire digit. At the affected site, a swelling was associated with a glassy and tender skin (Fig. 1a). Edema in pigs was commonly associated with heavy intensity of sand flea infections but in dogs it was detected even at sites with a single lesion. Skin ulcerations which occurred in association with hoof wall erosions always occurred in association with heavy infections and extensive necrosis. Necrosis mostly involved the skin but in intense infections it extended to the hoof wall proximal to the coronary band (Fig. 1b), the major predilection site. As a rule, sites of heavy infection presented also with mutilated lesions especially on the hoof bulb and sometimes on cranial and lateral sides of the digits. Extensive hoof wall necrosis resulted in detachment of the horny wall from the digits at various points (Fig. 1b) and in one severely affected pig; the horny walls of four dew claws from three different legs (two hind and one front leg) were entirely lost (Fig. 1c).

Deep fissures were most frequent at infection sites located at the skin-hoof wall junction (Fig. 1d) among pigs with variable intensity. The lateral and the cranial aspects of the digits frequently showed hyperkeratosis which was characterized by skin thickening, folds and alopecia (Fig. 1d). Hoof wall erosions also occurred either on the cranial or lateral aspect of the hoof or both (Fig. 1d). A single pig presented an abscess where a sand flea was embedded on the coronary band of the lateral principal digit of a leg which had been tightly tied with a rope.

Pain was detected when pressure was applied on lesions and surrounding sites of pigs and puppies. Resentment or reaction to palpation was evidenced by limb retraction when the affected site was palpated. For adult dogs it was usually not possible to reliably ascertain if the infection was associated with pain, since most of them had to be sedated for investigations. Hyperemia occurred as red rims of variable intensity and size around individual lesions but some coalesced at heavily infected sites. Hyperemia was generally most prominent in animal body parts with light skin (Fig. 1e, f) and at sites close to the sole of the digits (e.g. hoof bulb in pigs) where features of mutilation of lesions were also evident. Rubbing of infected sites on legs against the ground or the body trunk was observed in five (4.1 %) pigs while lesion mutilation by bites was evident in three (15 %) dogs. The biting of dogs on sand flea lesions resulted in exteriorization of embedded sand fleas (Fig. 2a). Figure 2b shows a hyperaemic lesion at a foot of a dog.

Edema, hyperemia, pain, ulcerations, fissures and abscesses were considered to be acute presentations while hyperkeratosis, necrosis, hoof deformity, erosions, roughening, loosening and loss of claws were assumed to be due to chronic infections. Overall, both acute and chronic pathology frequently coexisted. A higher proportion of infected dogs (80 %) presented with acute features of tungiasis than pigs (47.9 %; *p* = 0.001) while more pigs exhibited chronic clinical pathology than dogs (68.6 % vs. 30 %; *p* < 0.0002).

### Effect of tungiasis on body weight and body condition scores

Apparently there was no correlation between body condition score and intensity of infection in both pigs (rho = −0.058; *p* = 0.5) and dogs (rho = −0.34; *p* = 0.14). Body weight and body condition scores never differed significantly among pigs with variable intensity of infection (Table 4).

**Table 4:** Comparison of intensity of infection with body condition scores and live body weight of infected pigs

Variable:	Intensities of infection compared		*p*-value
	Low (1–4 lesions), *n* = 47	Medium (5–30), *n* = 44	
	Low (1–4 lesions), *n* = 47	Medium (5–30), *n* = 44	
Age in months: median (range)	5 (0.5–30)	5.5 (0.5–30)	0.35
Number of lesions: median (range)	2 (1–4)	10 (5–30)	*p* < 0.0001
Body condition score (scale of 1–5): median (range)	3 (2–4.5)	3 (2.5–4)	0.31
Estimated live body weight in kg: median (range)	25 (2.5–106)	15 (3–110)	0.1
	Low (1–4 lesions)	Heavy (>30 lesions), *n* = 30	
Age in months: median (range)	5 (0.5–30)	5.5 (2–18)	0.7
Number of lesions: median (range)	2 (1–4)	79 (38–246)	*p* < 0.0001
Body condition score (scale of 1–5): median (range)	3 (2–4.5)	3 (2.5–5)	0.52
Estimated live body weight in kg: median (range)	25 (2.5–106)	35 (3–104)	0.47
	Medium (5–30 lesions)	Heavy (>30 lesions)	
Age in months: median (range)	5.5 (0.5–30)	5.5 (2–18)	0.18
Number of lesions: median (range)	10 (5–30)	79 (38–246)	*p* < 0.0001
Body condition score (scale of 1–5): median (range)	3 (2.5–4)	3 (2.5–5)	0.81
Estimated live body weight in kg: median (range)	15 (3–110)	35 (3–104)	0.08

### Other findings

Other types of ectoparasites were detected among 111 (91.7 %) pigs and in all of the 20 (100 %) dogs with tungiasis. Up to three other types of ectoparasites were found on dogs. These included hard ticks of various species (*n* = 16, 80 %), mites causing mange (*n* = 3, 15 %) and other species of non burrowing fleas (*n* = 17, 85 %). Similarly, three other types of ectoparasites were found on infected pigs. These included pig lice (*n* = 106, 87.6 %), different species of ticks of the family Ixodidae (*n* = 53, 43.8 %) and mange mites (*n* = 13, 10.7 %).

## Discussion

Hitherto, there are only a few case series but no systematic studies on tungiasis-associated morbidity in animals [5, 14, 17, 19, 20, 25]. The occurrence of tungiasis is widely unknown among both, farmers and animal health workers, and its health significance is widely underestimated. This study presents the first systematic description of tungiasis-associated clinical pathology in pigs and dogs. Infected pigs came from nine villages while dogs were from five villages in a typical rural setting in Uganda. Therefore the findings in the present study can be considered to be representative of rural Uganda and probably East Africa. In most tungiasis-endemic regions, either pigs or dogs are the most frequently affected domestic animals and harbor high parasite loads [5, 13, 15, 17]. Since the severity of tungiasis strongly correlates with parasite load, it was expected that these species show severe morbidity [10, 11].

Sand fleas are obligatory haematophagous parasites and preferentially penetrate soft and well vascularised lower body parts [9]. Indeed all lesions in infected dogs and the majority of lesions in pigs (99.3 %) were located on the limbs. Localization of sand fleas predominantly on feet is not unique to dogs and pigs but this is also the case in most susceptible animal species [26] and humans [10, 27]. This has been attributed to the small size and poor jumping ability of sand fleas [9]. Unlike humans, where sand fleas localise randomly at various sites of the feet [10, 28, 29], this study has revealed that in the case of pigs and dogs, sand fleas on feet preferentially embed at claw coronary bands and nail basement, respectively. Similar to Wistar rats [26], both pigs and dogs, had more lesions on hind than front legs.

It is unknown how much blood an embedded female sand flea consumes during the vital stages 1 to 3. It is conceivable, though, that with a high parasite load and cumulative parasite burden during the year, infected animals can become anaemic. In fact, five piglets had discernible features of anaemia but none of the infected dogs exhibited gross anaemia. It was impossible to attribute the anaemia to tungiasis as in the study area with poor husbandry practices, anaemia is usually multi-factorial.

Earlier reports suggested that ectopic localization of sand fleas are quite common in animals [13, 17–19]. However, these reports were based on a few pigs either from the abattoir or individual households. In humans, occurrence of lesion ectopy has been shown to increase with parasite load [28, 30]. In this study, ectopic lesions were only detected in two pigs (1.7 %) of the 121 pigs with tungiasis and less than 1 % of the total number of lesions were located on the scrotum and the perineum. Similar to humans, pigs with sand flea ectopy had a high parasite load (30 and 45 lesions, respectively). Sand flea lesions have been observed in locations other than legs, i.e. in pigs on the mammary glands, snout and the tail [5, 17–20] and in dogs on the muzzle [13] and mammary glands [31].

Although, Cooper [19] did not detect tungiasis-associated pruritus among *T. penetrans* infected pigs, we detected five pigs which were scratching infected sites against the ground or other body parts. The erosions seen on the skin and on the hoof wall at sites with lesions were also corroborating evidence for itchy lesions that provoke rubbing of affected sites against other surfaces. In addition, many lesions were partially defaced which is also an indirect feature of itching. Mutilation of lesions among infected animals has been reported before [5] although not clearly associated with itchy lesions. Among infected dogs, three had exteriorised sand fleas with bite marks; features which also suggest pruritus and/or pain of the lesions.

Congestion around lesions appeared more intense at sites that had been rubbed against other surfaces hence the inflammatory changes appeared to have been intensified by lesion scratching in pigs. Bacterial super infections of lesions are very frequent in humans [32] and they amplify the inflammation. A few case reports indicate secondary bacterial infections in animal tungiasis eventually leading to micro abscesses and septicaemia [5, 13, 17]. In the present study only one pig had a discernible abscess and neither any of the infected dogs nor pigs had fever which might indicate septicaemia.

Ulcers were common among both infected pigs and dogs but fissures were only detected in pigs. Fissures were mostly seen at the junction of the skin and the horny hoof wall where the hypertrophic sand flea had detached the skin from the hoof. This is similar to the location of fissures in humans [10]. Whereas in humans, fissures were associated with a high intensity of infection [10], in this study they were detected in pigs with variable infection intensities.

Pigs which had problems of using their legs; concomitantly had features of chronic infections such as severe tissue necrosis with loosening of the digital hooves. However, many heavily infected pigs did not show any obvious signs of tungiasis-associated ill-health, an observation reported before [19, 20]. Severe morbidity with economically significant sequelae have been reported before among pigs. These include disabling limb deformities; painful snout and teat infections manifesting with agalactia, anorexia and mastitis in suckling sows resulting in piglet starvation [5, 17, 18]. This study has reported the loss of horny hoof wall in pigs for the first time.

Although *T. penetrans* infections caused lesions with significant effects on pig health, the body weight and condition scores of those infected apparently never changed with intensity of the infection. Since the pigs were from poor rural communities where there is minimum regard to good husbandry practices, other factors such as nutrition and helminthiasis are possible cofounders of the observations. More controlled studies are required to establish the effect of tungiasis on the general health and therefore productivity of the pigs.

Given the fact that more pigs had lesions on hind legs and principal digits than front legs and accessory claws, for rapid detection of tungiasis in pigs, coronary bands of principal digits of hind legs should be examined. Principal digits offer a larger surface for sand flea attachment and are closer to the ground than accessory digits hence the preferential sand flea attachment. For the same reason, more dogs had lesions on the digital foot pads and they also had more lesions than other sites hence rapid assessments of dogs for tungiasis should focus on their claw beds.

Clinical pathology due to tungiasis in pigs and dogs may mimic other conditions from which they should be distinguished. Such include myiasis, focal horny cutaneous growths, small abscesses, pox lesions, vesicular diseases, mastitis, mange, mycoses, foreign body granulomas, warts and dermatitis [5, 33].

The high overall parasite load and the presence of a wide range of other ectoparasites indicate the absence of effective ectoparasites control in the communities studied. There is a need to demonstrate the economic significance of tungiasis and the role of *T. penetrans* in the transmission of pathogens. This may arouse more interest in the management of this rather neglected ectoparasitosis.

## Conclusions

This study presents the first systematic and comprehensive description of clinical tungiasis in the most important animal reservoir hosts under endemic conditions in Africa. The study has demonstrated occurrence of a wide range of structural and functional manifestations of *T. penetrans* infections in pigs and dogs closely resembling those described in humans and Wistar rats [10, 25] which warrant veterinary interventions. As a prerequisite for treatment of animal tungiasis, commercially available ectoparasiticides should be validated for tungicidal and *Tunga* prophylactic properties under field conditions. Chemical control should be integrated with other supportive strategies such as farmer education on proper animal husbandry practices and environmental sanitation.
